# Phenotypic and genotypic aspects of Townes-Brock syndrome: case report of patient in southern Brazil with a new SALL1 hotspot region nonsense mutation

**DOI:** 10.1186/s12881-017-0483-7

**Published:** 2017-11-06

**Authors:** Paulo Breno Noronha Liberalesso, Mara L. Cordeiro, Simone Carreiro Vieira Karuta, Karyn Regina Jordão Koladicz, Anderson Nitsche, Bianca Simone Zeigelboim, Salmo Raskin, Michael Rauchman

**Affiliations:** 1 Department of Neuropediatrics, Hospital Pequeno Príncipe, Curitiba, Parana Brazil; 2 Universidade Tuiuti do Paraná, Otoneurology Research Center, Curitiba, Parana Brazil; 3 Neurosciences Research Group, Pelé Pequeno Principe Research Institute, Curitiba, Brazil; 4 Faculdades Pequeno Principe, Curitiba, Brazil; 5 Department of Psychiatry and Biobehavioral Sciences of the David Geffen School of Medicine, Semel Institute for Neuroscience and Human Behavior, University of California, Los Angeles, USA; 6 Department of Medical Genetics, Hospital Pequeno Príncipe, Curitiba, Parana Brazil; 7 Group for Advanced Molecular Investigation (NIMA), Graduate Program in Health Science (PPGCS), Health and Biosciences School (ESB), Pontifícia Universidade Católica do Paraná (PUCPR), Curitiba, Parana Brazil; 8 Department of Internal Medicine (Nephrology), St. Louis University School of Medicine, and St. Louis VA Medical Center, St. Louis, USA

**Keywords:** Case report, Townes-Brock syndrome, Sall1 mutation, Pediatric

## Abstract

**Background:**

Townes-Brocks syndrome (TBS) is a rare autosomal dominant condition characterized by renal, anal, limb, and auditory abnormalities. TBS diagnosis can be challenging in settings where genetic analysis is not readily available. TBS traits overlap with those of Goldenhar and VACTERL syndromes.

**Case presentation:**

Here, we present the case of a 5-year-old Brazilian boy born with an anorectal abnormality, limb and external ears malformations, genitourinary anomalies, and a congenital heart defect. Genetic analysis revealed a *SALL1* nonsense mutation. The case is discussed in the context of the current literature.

**Conclusions:**

Because of the variability in TBS clinical presentation, genetic analysis is key to the differential diagnosis of TBS relative to phenotypically similar syndromes.

## Background

In 1972, Townes and Brocks [1] described a heritable syndrome, now known as Townes-Brocks syndrome (TBS), characterized by anal, hand, foot, and ear abnormalities—including an imperforate anus or anorectal malformation, supernumerary thumbs, external ear malformations, pre-auricular tags, and sensorineural hearing loss—in a father and five of his seven offspring. After this initial report, several cases were documented around the world [2–4]. The clinical presentation of TBS is highly variable, with intelligence and behavior being normal in most cases [5].

TBS is rare, with an estimated prevalence of 1:250,000 live births [6]. The diagnostic criteria for TBS are the presence of two or more of the following: (a) anorectal malformation (e.g., anal stenosis, imperforate anus, anteriorly situated anus, recto-vaginal or recto-urethral fistula); (b) hand malformation (e.g., polydactyly, bifid or triphalangeal thumb); (c) external ear malformation (e.g. preauricular tags or pits, external ear underdevelopment/microtia, or satyr ear) with sensorineural hearing loss; and (d) a family history that includes a relative diagnosed with TBS. Genitourinary anomalies such as renal hypoplasia/dysplasia, multi-cystic kidneys, and renal agenesis have been reported [7, 8], but their clinical and histopathological features have not been clarified. Although most congenital malformations appear to be clinically stereotyped, cases of phenotypic heterogeneity have been described [7, 8].

Because TBS is a polymorphic syndrome that can affect various organs and systems, it can be confused with other genetic syndromes, such as VACTERL association (vertebral anomalies, anal atresia, cardiovascular anomalies, tracheoesophageal fistula, renal and/or radial anomalies, limb defects) [3]. The presence of hearing loss with preauricular tags and/or satyr ears, however, can distinguish TBS, for which these traits are characteristic, from VACTERL association, for which they would be uncommon. On the other hand, vertebrae, trachea, esophagus, and radial bone abnormalities do not occur in TBS and are very common in VACTERL association [3]. Incomplete formation of the external ear also occurs in Goldenhar (oculo-auriculo-vertebral) syndrome [9]. However, Goldenhar syndrome can be differentiated from TBS by the presence of other clinical features, such as incomplete development of the nose, soft palate, lip, and mandible as well as the absence, in most cases, of upper limb or anal malformations, overlapping toes, and hypospadias [9].

TBS results from mutations of the developmental gene spalt-like transcription factor 1 (*SALL1*) gene [10]. The SALL1 protein is a putative zinc finger transcription factor related to the developmental regulator sal of *Drosophila melanogaster* that contains nine C2H2 zinc finger domains and one C2HC zinc finger domain, as well as glutamine-, proline-, alanine-, and serine-rich domains [6, 10]. TBS shows an autosomal dominant inheritance pattern, with the risk of inheriting TBS for children of affected parents being 50%. In sporadic cases, with non-affected parents, the recurrence risk in further pregnancies has been reported to be 1~5%; sporadic cases are attributed to de novo mutations or germline mosaicism in parents [6].

Here we report the case of a Brazilian child (VMFS) with TBS and discuss clinical and genetic aspects of the case.

## Case presentation

The proband, VMFS, who was 5 years old at the time this report was written, was born by normal vaginal delivery at 38.5 weeks gestational age with Apgar scores of 9 and 10 (first and fifth minutes, respectively). His birth weight (2.735 kg), length (48 cm), and head circumference (34.5 cm) were within normal ranges. The pregnancy occurred with no intercurrences and no concerns were raised in prenatal examinations. The child was born with anal atresia, which was corrected by posterior sagittal anorretoplasty on his second day of life. After the operation, VMFS remained hospitalized for 10 days in the neonatal intensive care unit.

VMFS is the second child of non-consanguineous healthy young adult parents. He has a brother (11 years old) with attention deficit hyperactivity disorder and a cousin who was born with an imperforate anus. The patient has exhibited developmental and speech delays. He displays hyperactive and sometimes aggressive behavior, which has been managed with antipsychotic drugs starting from the age of 4. He has a short stature for his age, a low anterior hairline, left microtia with agenesis of the helix, bilateral preauricular tags, low set ears, long eyelashes, epicanthus, a deviated nose with a downward pointing tip, and a short neck. Skeletal anomalies were prominent and included a bifid thumb on the right hand, a long left thumb with a size and shape similar to that of the second left hand finger, and overlapping toes on the feet. VFMS was also noted to have hypospadias and a hypochromic spot on the right thigh.

**Fig. 1:**
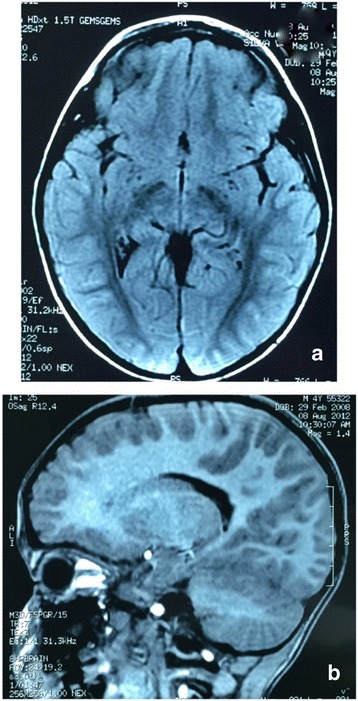
**a** and **b** *.* Coronal and sagittal views of brain computerized axial tomography and magnetic resonance imaging

**Fig. 2:**
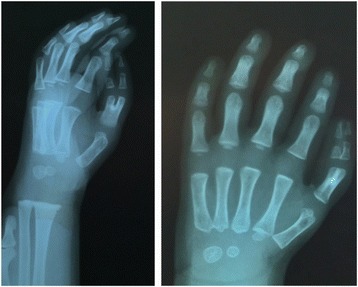
Right thumb of the patient shows preaxial polydactyly with ulnar deviation

An abdominal ultrasound was normal. Echocardiography showed an atrial septal defect. Cystourethrography showed vesicoureteral reflux (grade II) and bilateral reduction of the distal urethral caliber. However, blood tests results ruled out renal insufficiency, and renal scintigraphy and ultrasonography findings were normal. Analysis of auditory brainstem evoked potentials indicated bilateral moderate hearing, with better responses to low-frequency stimuli. Brain computerized axial tomography and magnetic resonance imaging scans were normal (Fig. 1). An electroencephalogram (International 10–20 System of Electrode Placement), showed normal background activity. X-rays showed preaxial polydactyly with ulnar deviation (Fig. 2), and a rudimentary rib at T12.

A karyotype performed with peripheral blood lymphocytes by the banding technique demonstrated a normal male chromosome constitution (46XY). DNA sequence analysis of the *CHD7* gene, responsible for CHARGE syndrome, showed no polymorphisms. This prompted sequencing of the *SALL1* gene, which identified a novel heterozygous nonsense mutation in the mutational “hotspot” of exon 2, a c.824 T > G (p.L275X).

Since his diagnosis at age of 2 years, VFMS has received continuous medical follow-up. He has been receiving physical therapy since the age of one year since he had signs of development delays, and speech therapy since the age of 2 years. He attends a special education school.

## Discussion and conclusion

We report the case of a boy, in which TBS was identified phenotypically and confirmed genotypically. This case is the first case of TBS to be reported from a major pediatric hospital in southern Brazil. The clinical aspects of the case are as follows:

### Anorectal malformation

VFMS was born with anal atresia. Anorectal abnormalities are present in most patients with TBS and represent the most common feature of the syndrome. In some patients, imperforate anus is associated with recto-perineal or recto-vaginal fistulas. In others, the anus is very anteriorly situated or surrounded by excess skin. Chronic constipation and gastroesophageal reflux are common [11].

### Limb malformation

VFMS was born with a bifid right thumb, three phalanges in his left thumb, and overlapping toes. Limb malformations are very common in TBS, with the most common presentations being polydactyly, hypoplastic and/or broad thumbs, tri-phalangial thumb (often in association with pre-axial polydactyly), and distal ulnar deviation and/or bifurcation of the thumbs. Although there have been reports of overlapping and syndactylous toes in patients with TBS, skeletal malformations of the lower limbs are infrequent in TBS. Hand radiographs may reveal malformed or absent triquetrum and/or navicular bones. The presence of more serious limb malformations, particularly involving the radius, are not characteristic of TBS [11, 12].

### External ear malformation with sensorineural hearing loss

VFMS’ ear malformations included left microtia with agenesis of the helix and bilateral pre-auricular tags. Additionally, he suffered from bilateral moderate hearing loss, with better responsivity to low-frequency stimuli. External ear malformations involving one or both ears, including small ears, over-folded superior helixes, and small anthelixes, are typical of TBS. Pre-auricular tags are also very common in TBS. Hearing loss is frequent, variable in severity, and classified most often as sensorineural affecting high frequencies [11, 12]. Some patients have mixed hearing loss (neurosensorial and conductive) due to ossicular anomalies (mainly of the hypoplastic malleal head) or an abnormally shaped oval window [1, 6].

### Genitourinary anomalies

VFMS has vesicoureteral reflux and a bilaterally reduced distal urethral caliber. Some patients with TBS have renal hypoplasia, and unilateral renal agenesis or dysplasia have been reported rarely in TBS [12]. Mutations in *Sall1* cause severe renal hypoplasia or ageneis in mice [13, 14]. Although vesicoureteral reflux has not been observed in mouse models, improper separation of the developing ureter from the common nephric duct has been observed [14]. Compromised kidney function may occur with or without structural abnormalities (e.g., polycystic kidneys, malrotation, ectopia, horseshoe kidney, or hypoplasia). Chronic renal failure, renal hypodysplasia, and focal segmental glomerulosclerosis have been described in adults with TBS. Cryptorchidism, hypospadias, vaginal aplasia in association with a bifid uterus, and bifid scrotum occur in TBS with variable incidence [15, 16].

### Congenital heart defects

Echocardiography revealed an atrial septal defect in VFMS. Although the presence of a congenital heart abnormality is not part of the diagnostic criteria for TBS, about 25% of cases involve a congenital heart abnormality, such as truncus arteriosus, ventricular septal defect, pulmonary atresia, and atrial septal defect [12, 17].

### General manifestations

Formal psychometric and intelligence analysis tests were not applied in the present case because of the young age of our patient. However, signs of a behavioral disorder were evident, including psychomotor agitation and occasional bouts of aggression. At 4 years of age, VFMS’ weight and height are below the normal ranges for his age.

Although most patients with TBS described in the literature have been reported to be of normal intelligence, mild or moderate mental retardation occurs in approximately 10% of cases, 3- to 4-fold the rate of mental retardation in the general population [5]. Behavioral disturbances are also considered to be more frequent in children with TBS than in the general population [11]. The incidence of growth retardation is not known. However, a third of the TBS cases in the literature involve some degree of postnatal growth retardation [11, 12, 17]. Although central nervous system abnormalities are not described as a frequent characteristic of the syndrome, Harrison et al. [18] found that brain neurons are particularly sensitive to *Sall1* mutations. In animal models, *Sall1* gene deletion results in reduced thickness of the cerebral cortex during embryonic development. These structural changes may compromise cognitive development. However, homozygous *SALL1* mutations were reported recently in two patients with severe central nervous system defects, including hypoplastic corpus callosum, further indicating an important role for *Sall1* in brain development [19].

### Family history

The presence of a relative with TBS is considered a potential diagnostic criterion [3, 7]. VFMS had only a cousin with an imperforate anus. Because only the proband was subjected to genetic testing, we do not know whether the present case was familial or sporadic.

### Molecular genetics

The *SALL1* gene was mapped to chromosome 16q12.1, originally by Kohlhase et al. [20] with subsequent confirmation by Chai et al. [21]. At least 60 mutations in the *SALL1* gene have been described in patients with TBS [21]. The *spalt* (*sal*) family of genes were identified initially because their mutation elicited transformations in the tail and head of the Drosophila embryo. *SALL1* contains three exons and two introns, and all published mutations are located in exon two or intron two. SALL genes, which encode for zinc finger-containing transcription factors, are highly expressed during embryogenesis and play fundamental roles in animal development. *SALL1* encodes a 1306-amino-acid protein that is expressed mainly in brain, liver, and kidney [21–23].

In 1999, Kohlhase et al. [10] examined 23 families with TBS or a TBS-like syndrome for *SALL1* mutations with the aim of establishing genotype-phenotype correlations. Mutations were identified in 11 families, all located 5′ of the first double zinc finger-encoding region. However, the authors were unable to link particular phenotypic characteristics of the syndrome to mutation sites within the gene. VFMS has a nonsense mutation in the mutational “hotspot” of exon 2, exactly one codon before the most common mutation, p.Arg276X. Typically, nonsense-mediated decay (NMD) prevents the accumulation of abnormal proteins. However, at least two *SALL1* mutations, including p.Arg276X, escape NMD [24]. Truncated mutant SALL1 proteins that escape NMD do not result in haploinsufficiency, but rather produce dominant effects that cause TBS phenotypes [14, 24]. Interestingly, other SALL1 forms with mutations near Arg276 are subject to NMD [25]. There appears to be a sharp boundary distinguishing NMD-activating from NMD-bypassing mutations related to the transcript’s ability to reinitiate translation from a cryptic downstream start codon [25]. Because dominant negative versus haploinsufficient mechanisms may underlie phenotypic variability, delineation of NMD-activating versus NMD-bypassing mutations is important for further genotype-phenotype interpretation [22].

Nonsense mutations are usually associated with classical TBS and a more severe phenotype, including renal failure [26]. In the patient here reported, meanwhile, Cystourethrography showed vesicoureteral reflux (grade II) and bilateral reduction of the distal urethral caliber. However, blood tests result still do not show renal insufficiency. Therefore, follow-up of renal function is one of the most important anticipatory guidance measures in this patient.

In conclusion, VFMS had classical TBS, which was confirmed by molecular genetic analysis. A careful analysis of the phenotype is essential for differentiation of TBS from other syndromes, such as Goldenhar and VACTERL association. Knowledge of phenotypic and genotypic aspects can facilitate early diagnosis and treatment has an impact on prognosis, and allows more effective genetic counseling and prenatal diagnosis for the patient and his relatives.

## Abbreviations

TBS: Townes-Brock syndrome
VACTERL: V: Vertebral anomalies, A: Anal malformation, C: Cardiovascular defect, TE: Tracheal and esophageal malformation, R: Renal agenesis, L: Limb abnormalities
